# Long‐term shifts in pikeperch, *Sander lucioperca* , diet composition in a changing reservoir

**DOI:** 10.1111/jfb.70225

**Published:** 2025-09-17

**Authors:** Karlos Ribeiro de Moraes, Million Tesfaye, Daniel Bartoň, Petr Blabolil, Jakub Brabec, Marek Brabec, Vladislav Draštík, Marie Prchalová, Josef Matěna, Milan Muška, Michal Tušer, Tomáš Jůza, Luboš Kočvara, Zuzana Sajdlová, Katerina Soukalová, Allan T. Souza, Marek Šmejkal, Radka Symonová, Milan Říha, Mojmír Vašek, Jan Kubečka

**Affiliations:** ^1^ Biology Centre of the Czech Academy of Sciences Institute of Hydrobiology České Budějovice Czech Republic; ^2^ Faculty of Fisheries and Protection of Waters, South Bohemian Research Centre for Aquaculture and Biodiversity of Hydrocenoses University of South Bohemia in České Budějovice Vodňany Czech Republic; ^3^ Faculty of Science University of South Bohemia České Budějovice Czech Republic; ^4^ Institute of Computer Science Czech Academy of Sciences Prague Czech Republic; ^5^ Institute for Atmospheric and Earth System Research INAR, Forest Sciences, Faculty of Agriculture and Forestry, University of Helsinki Helsinki Finland

**Keywords:** cannibalism, diet shift, prey–predator ratio, stomach content

## Abstract

As a significant piscivorous predator, the pikeperch (*Sander lucioperca*) plays a critical role in regulating aquatic ecosystems. Understanding its size‐related feeding behaviour is thus vital for assessing its impact. To evaluate its role in the ecosystem, stomach content analysis provides useful insights into predator–prey dynamics. This study investigated the stomach content and dietary patterns of pikeperch in the Lipno Reservoir, Czech Republic, across two distinct time periods: the 1966–1970 (19's) and the 2010–2022 (20's). We analysed the stomach contents of 875 pikeperch specimens and revealed a notable shift in diet composition over time. The results demonstrated a significant decrease in overall prey consumption and a marked increase in the proportion of cyprinids in the diet, whereas the percentages of perch (*Perca fluviatilis*) and ruffe (*Gymnocephalus cernua*) decreased. Cannibalism was observed in the 20's dataset, suggesting a reduction in prey abundance in the reservoir or a shift in community structure. The predator–prey length ratio (PPR) decreased as pikeperch grew larger. The PPR values in Lipno Reservoir were lower than those reported in other studies, indicating potentially slower growth rates in this fish. Although percids were the main prey of pikeperch, the electivity index showed positive values only for pikeperch as the prey. Overall, this study highlights long‐term changes in pikeperch diets, both in the numbers and species of prey and in PPR.

## 1 INTRODUCTION

Pikeperch, *Sander lucioperca* Linnaeus, 1758, is a large‐bodied piscivorous fish from freshwater and brackish water habitats in western Eurasia and is important for both recreational and commercial fisheries (Lappalainen et al., 2003). As a top predator, the pikeperch plays important ecological and regulatory roles in aquatic communities (Kopp et al., 2009). Pikeperch is routinely stocked across European freshwater systems for two primary objectives: to compensate for declining natural recruitment and maintain viable populations in anthropogenically altered habitats (e.g., dammed rivers, overfished lakes), and to exert top‐down control on forage fish stocks (e.g., roach *Rutilus rutilus*, bleak *Alburnus alburnus*), thereby mitigating ecological imbalances caused by prey overabundance (Jakubavičiūtė et al., 2024; Rita et al., 1996). Pikeperch is a piscivorous ambush‐pursuit solitary predator inhabiting both benthic and pelagic zones. As opportunistic feeders, their prey selection primarily depends on two factors: the current abundance of available fish species and the size range of potential prey fish in their habitat (Anders, 2001).

Body size acts as a trait governing ecological processes across biological scales (Woodward et al., 2005). At the individual level, size directly determines metabolic rates (Atkinson & Hirst, 2007; Cruz‐Font et al., 2019), prey selection thresholds (Bland et al., 2019; Turesson et al., 2002), foraging efficiency and predator–prey interaction rates (Martins et al., 2023; Stephens & Krebs, 1986). These size‐dependent mechanisms scale up to shape population biomass distributions and food web architecture (Woodward et al., 2005), creating linkages between individual physiology and ecosystem patterns (Bland et al., 2019; Martins et al., 2023). Extending body‐size effects from individuals to populations and communities could enhance our understanding of ecological patterns and processes across these levels (Martins et al., 2023; Woodward et al., 2005). The assumption that the body‐size effect at an individual level may be scaled up to the species level has provided new insights into how food web structure and dynamics are constrained and associated with predator and prey body sizes (Bland et al., 2019; Woodward et al., 2005). The concept of ‘predator window’, introduced by Claessen et al. (2002), discusses how the success of predation is linked to the predator–prey length ratio. This study indicates that the lower size threshold of the predation window influences population dynamics by regulating prey recruitment success and potentially boosting cannibalistic interactions.

Stomach content analysis remains the primary method for evaluating such size‐structured interactions (Buckland et al., 2017; Ng et al., 2021). Such diet analyses are particularly valuable for tracking temporal and environmental changes in trophic structure (Jacobsen et al., 2023) and offering insights into community‐level interactions. In recent years, other methods have been used for fish diet analyses, such as isotope analysis (radio or stable), fatty acid analysis, non‐lethal extractions (tubing, induced regurgitation or forceps) or direct observations (Braga et al., 2012). However, dissection and stomach content analysis remain among the most widely used, cost‐effective and direct methodologies used for diet studies (Baker et al., 2014). This approach requires minimal instrumentation, allows for direct identification of ingested prey, while affording researchers a direct and precise quantitation and identification of dietary components (Braga et al., 2012; Carreon‐Martinez & Heath, 2010; Manko, 2016).

The aim of this study was to determine differences in the stomach contents of pikeperch in relation to fish community composition over time in the Lipno Reservoir, Czech Republic. Particularly we were interested in assessing the mean prey consumption rates, the relationship between predator and prey size and the species selectivity of pikeperch. We hypothesized that (i) the abundance and diversity of prey that pikeperch feeds on vary across different time periods (utilizing data coming from research campaigns decades apart); (ii) there is a relationship between predator size and prey size in pikeperch; and (iii) pikeperch exhibits species‐specific prey selectivity in their feeding behaviour.

## 2 MATERIALS AND METHODS

### 2.1 Study area

The Lipno Reservoir was built in 1960 as a hydropower reservoir, and has since transformed into a multipurpose system supporting flood control, water resource management and recreational use. The reservoir is a dam impoundment near the border with Austria on the Vltava River in the foothills of the Šumava Mountains. It is located in Southern Bohemia, Czech Republic. The reservoir has a volume of 306 million m^3^, a surface area of 46.5 km^2^, a maximum depth of 22 m and a mean depth of 6.6 m. The Lipno Reservoir was known for its productive pikeperch fishery for decades (Jůza et al., 2023; Vostradovska & Vostradovsky, 1986). However, after 2004, the catches declined by almost 90%, and the growth rate of pikeperch dropped sharply, with individuals reaching legal size 1 to 2 years later (Tesfaye et al., 2023).

### 2.2 Sampling

**FIGURE 1:**
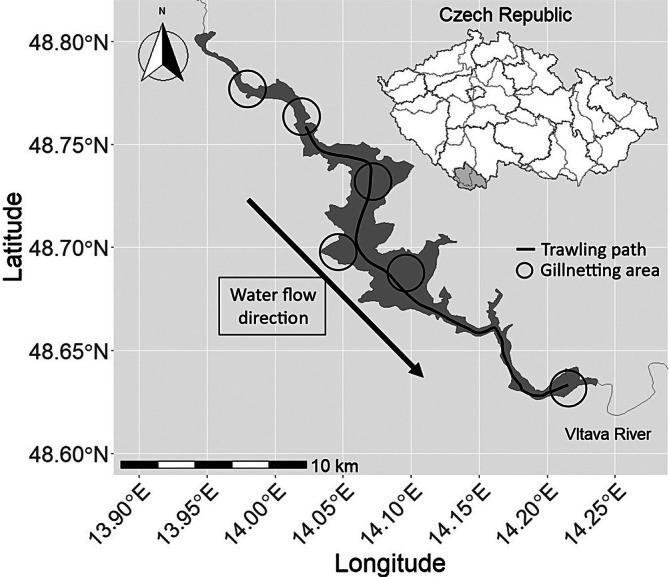
Lipno Reservoir with its location within the Czech Republic and the main sampling areas.

Pikeperch were sampled in Lipno Reservoir over two periods (Figure 1):

1. During 1966–1970, pikeperch catches came from the commercial fishing using seining and gillnetting, as described by Vostradovsky and Tichy (1999). The sampling period spanned July to September. The gillnets were exposed from the evening until the morning.
2. During 2010, 2012, 2016–2022, the pikeperch were sampled during regular annual standard surveys using two main methods (each year during the last week of August):
  2.1 European standard gillnet (ESG) (CEN, 2015) and large mesh gillnet (LMG) (Šmejkal et al., 2015). Sampling from 2010 to 2022 in Lipno Reservoir followed the European standard document (ESG) (the benthic gillnets: 1.5 m height × 30 m length, 2.5 m panels for each of 12 mesh‐sizes; the pelagic gillnets: 3 m height × 30 m length, 2.5 m panels for each 12 mesh‐sizes). The ESG mesh‐sizes followed a geometric series with a ratio of approximately 1.25 (5, 6.25, 8, 10, 12.5, 15.5, 19.5, 24, 29, 35, 43 and 55 mm). LMG consisted of four mesh‐sizes following the ESG geometric series (70, 90, 110 and 135 mm; knot to knot). Two net configurations of LMG were deployed: the pelagic nets (3 m height × 40 m length) and the benthic nets (1.5 m height × 40 m length), each comprising four 10‐m panels corresponding to different mesh‐sizes. Both net types were deployed concurrently with the ESG in identical habitats and locations. The nets were deployed at six main reservoir sections along the gradient from the tributary to the dam (Kubečka et al., 2022). The gillnets were exposed from the evening until the morning.

2.2. Night‐time pelagic trawling was employed to target species during their surface‐oriented feeding phase. Two different types of trawls were used: ‘fry’ trawl (3 m height × 3 m width, Jůza et al., 2023; Jůza & Kubečka, 2007) and ‘adult’ (8 m height × 18 m width, Říha et al., 2012). Each codend was equipped with a funnel to prevent fish from escaping. Trawling was performed at night in the ranges of 0–3, 3–6, 6–9 m and 0–8 m, respectively. Buoys and weights were used to keep the frame in position for the catch, and the trawl was dragged 100 m behind the towing boats. The tow was performed for 10–20 min at a speed of 0.8–1.1 ms^−1^.

During the second study period (2010, 2012, 2016–2022), we expanded our sampling to include young‐of‐the‐year (YOY) fish communities, given their predominant representation in pikeperch diet. YOY abundance was assessed using two complementary methods: pelagic YOY were sampled via fry trawling (see above), whereas littoral YOY were quantified using night‐time beach seining. The beach seine measured 10 × 3 m (length × depth) with 1.4 mm mesh‐size. Annually, we conducted 40 standardized seining stations distributed evenly along the reservoir's entire longitudinal axis, spanning from the dam to the tributary inlet. We calculated catch per unit of effort (CPUE) by standardizing each seine haul to an effective sampling area of 120 m^2^ and extrapolating abundance to individuals per hectare; see Kubečka et al. (2022) for more information. To precisely determine the overall density of the YOYs in the reservoir, we considered the volume of each pelagic layer and of the littoral habitat (VOST method, Tesfaye et al., 2022) and estimated YOY abundance as the number of individuals per reservoir hectare. Overall weighted YOY abundance was used for the proportional abundance of specific prey in the reservoir.

The pikeperch individuals were euthanized by spinal cord interruption. The field sampling and experimental protocols used in this study were performed in accordance with the guidelines and permission from the Experimental Animal Welfare Commission under the Ministry of Environment of the Czech Republic (reference no.: CZ 01679). The Experimental Animal Welfare Commission of Biology Centre of the Czech Academy of Sciences approved methods and ethics of the study. The standard length (SL) of pikeperch was measured to the nearest millimetre. The fish were dissected, and their stomachs were removed and placed in 4% formalin. In the laboratory, stomach contents were identified to the lowest possible taxonomic level (if possible), and its SL was measured (nearest millimetre, if the body was whole).

### 2.3 Data analyses

During our data analyses, the years from 1966 to 1970 are described as the 19's dataset, and the years 2010, 2012, 2016 and 2022 are described as the 20's dataset. Therefore, in the model equations below, we use dataset factor with two levels (19 and 20).

Generalized linear models (GLMs) with a quasi‐Poisson family with the log link were used to analyse differences in the mean number of preys per pikeperch (mean prey) across datasets (Equation 1) at the total catch level. Prey count data exhibited positive skewness and overdispersion (variance > mean), making the quasi‐Poisson model more appropriate than a standard Poisson GLM. For predator–prey length ratios, we fitted a Gaussian (normal) distribution GLM using a model that is quadratic in predator length.

(1) $$\text{𝒍𝒊𝒏𝒆𝒂𝒓 𝒑𝒓𝒆𝒅𝒊𝒄𝒕𝒐𝒓 𝒇𝒐𝒓 𝒒𝒖𝒂𝒔𝒊‐𝑷𝒐𝒊𝒔𝒔𝒐𝒏 𝑮𝑳𝑴∶}\;\log\;({\text{m𝒆𝒂𝒏}}_{\text{prey}})\sim \text{d𝒂𝒕𝒂𝒔𝒆𝒕}$$

The predator–prey length ratio (PPR) in our study was used to highlight the relation of size preferences or availability. This value was calculated using Equation (2).

(2) $$\text{𝑷𝑷𝑹}={\text{𝑺𝑳}}_{\text{prey}}/{\text{𝑺𝑳}}_{\text{predator}}$$

The PPR was calculated for all individual prey items and modelled to calculate the change in relation to the predator size and the difference between the datasets, following model Equation (3) fitted separately for the 19's and 20's datasets.

(3) $$\text{L𝒊𝒏𝒆𝒂𝒓 𝒑𝒓𝒆𝒅𝒊𝒄𝒕𝒐𝒓 𝒇𝒐𝒓 𝒈𝒂𝒖𝒔𝒔𝒊𝒂𝒏}\;\mathrm{GLM}\;\text{𝑷𝑷𝑹}\sim a+b\times {\text{𝑺𝑳}}_{\text{predator}}+c\times {\mathrm{SL}}_{\text{predator}}^{2}$$

where ‘*a*’ represents the intercept, ‘*b*’ represents the coefficient of linear component and ‘*c*’ represents the coefficient of quadratic component.

We compared our data with other studies, where we used WebPlotDigitizer (Rohatgi, 2024) to extract the information from the papers and model these data against our data. Studies by Bousseba_2020 (Bousseba et al., 2020, Morocco), Dörner_2007 (Dörner et al., 2007, Germany), Keskinen_2004 (Keskinen & Marjomäki, 2004, Finland) and Nolan_2018 (Nolan & Britton, 2018, England) were selected for this comparison. Due to the different types of measurement of the fish length in other studies, total length (Bousseba_2020, Dörner_2007 and Keskinen_2004) and fork length (Nolan_2018) were converted to SL using the length‐to‐length conversion formula by Binohlan et al., 2011 (Equation 4):

(4) Standard length=a+b×known length

where ‘*a’* (intercept) and ‘*b’* (slope) were extracted from Fishbase database, 0.0 and 0.825 for fork length and 0.0 and 0.786 for total length (Froese and Pauly, 2024).

To quantitatively compare size‐based predation patterns between our dataset and previous studies, we implemented a Gaussian GLM quadratic in predator's SL (Equation 5), stratifying on study (to allow for possibly different quadratic relationships for different locations/studies).

(5) $$\text{L𝒊𝒏𝒆𝒂𝒓 𝒑𝒓𝒆𝒅𝒊𝒄𝒕𝒐𝒓 𝒇𝒐𝒓 𝒈𝒂𝒖𝒔𝒔𝒊𝒂𝒏}\;\mathrm{GLM}:\text{𝑷𝑷𝑹}\sim a+b\times {\text{𝑺𝑳}}_{\text{predator}}+c\times {\mathrm{SL}}_{\text{predator}}^{2}$$

Description for this equation follows the explanation of the Equation (3).

Selective feeding in Lipno Reservoir was evaluated using the Kimmerer electivity index (*Xi*) (Kimmerer & Slaughter, 2021) as follows:

(6) $$X_{i}=O_{i}/\left(1+O_{i}\right)$$

where *Oi* is obtained from the Jacobs’ *Q* index (Jacobs, 1974):

(7) $$O_{i}=g_{i}\;\left(1–a_{i}\right)/\left[a_{i}\;\left(1–g_{i}\right)\right]$$

where *g*_*i*_ is the proportional abundance of specific prey in the stomach, and *a*_*i*_ is the proportional abundance of specific prey in the reservoir. This index varies from 0 to 1, where 0.5 would be the similar ratio (no selection), a higher *X*_*i*_ value means a positive prey selection, and a lower *X*_*i*_ represents a negative prey selection. The year 2019 was excluded from this index, because only one species was found in the stomachs of pikeperch (proper calculation requires at least two species). This index was only calculated for the 20's dataset, where the data of the total YOY fish abundance were available; see the Sampling section earlier.

Cyprinid prey was excluded from family‐level analyses due to their low presence in the stomachs. The stats and mgcv packages were used to compute the GLMs. ggglot2 library was used for plotting. All data analyses were performed using R software (R Core Team, 2024).

## 3 RESULTS

Of the 875 pikeperch specimens analysed in this experiment, 599 had fish or fish remains in their stomachs. The 19's dataset comprised 435 pikeperch specimens with a mean SL ± standard error (SE) of 236.75 ± 3.3 (range: 69–660 mm). The 20's dataset comprised 440 pikeperch specimens with the mean SL ± SE of 284.46 ± 9.75 (range: 105–620 mm) (Table 1).

**TABLE 1:** Stomach sample summary by year, showing the number of empty stomachs, stomachs containing fish, total stomachs sampled and the proportion of empty stomachs.

Year	Empty	With fish	Total	Empty proportion
1966	3	30	33	0.091
1967	7	38	45	0.156
1968	26	174	200	0.130
1969	34	95	129	0.264
1970	5	23	28	0.179
2010	39	55	94	0.415
2012	33	7	40	0.825
2016	27	26	53	0.509
2017	30	64	94	0.319
2018	5	8	13	0.385
2019	5	1	6	0.833
2020	35	44	79	0.443
2021	7	3	10	0.700
2022	20	31	51	0.392
Total	276	599	875	0.315

**FIGURE 2:**
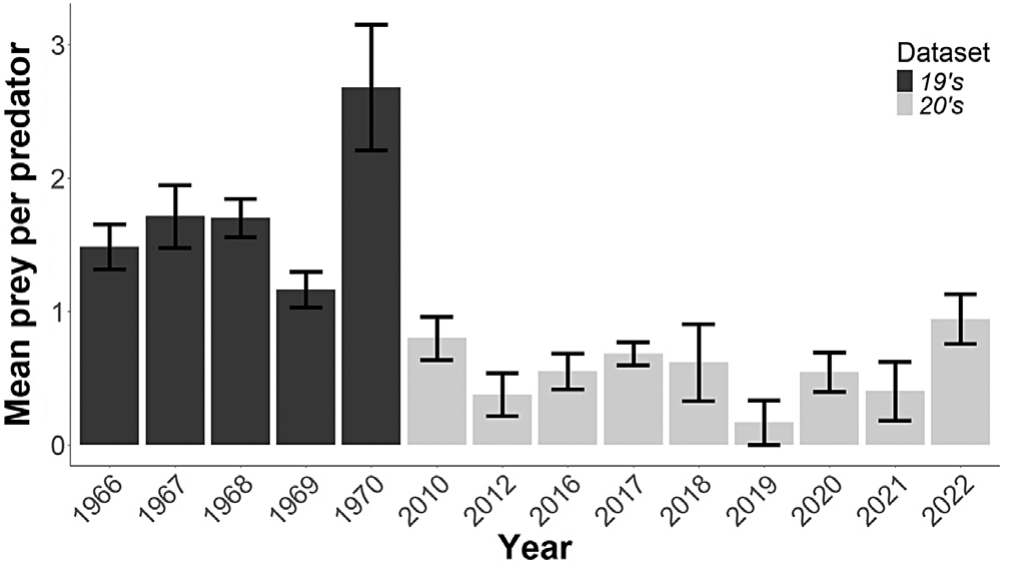
Interannual variation in the mean number of fish prey items per pikeperch. Values are derived from the analysis of all stomachs. Error bars indicate ± value of the standard error of the mean.

The conditional mean number of preys per pikeperch differed significantly between time periods – [GLM quasi‐Poisson, degree of freedom (df) = 12, *R* ^2^ = 0.74, *t* value = −5.727, *p* < 0.0001]; the values were 1.66 in the 19's and 1.16 in the 20's datasets (Figure 2).

Four prey species were identified in the stomach contents of pikeperch during our study in the 19's dataset: 86.93% perch (*Perca fiuviatilis*), 12.33% ruffe (*Gymnocephalus cernua*), 0.59% roach (*R. rutilus*) and 0.15% silver bream (*Blicca bjoerkna*). Six prey species were identified in the stomach contents of pikeperch in the 20's dataset: 41.24% perch, 39.78% pikeperch, 9.49% roach, 6.57% ruffe, 2.19% bleak (*A. alburnus*) and 0.73% bream (*Abramis brama*).

**FIGURE 3:**
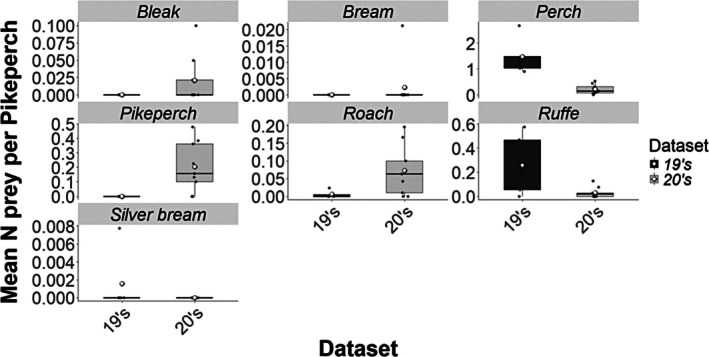
Mean prey‐per‐pikeperch specimen separated by species between datasets. Median values (thick lines), upper and lower quartiles (boxes), maximum and minimum values (whiskers) and outliers (dots) are shown.

Analysis of stomach contents by prey species revealed significant shifts in prey‐per‐predator ratios between the 19's and 20's datasets, reflecting changes in both prey composition and numerical abundance per pikeperch. In the 19's, perch (GLM quasi‐Poisson, df = 12, *R* ^2^ = 0.74, *t* value = −5.078, *p* = 0.0003) and ruffe (GLM quasi‐Poisson, df = 12, *R* ^2^ = 0.46, *t* value = −2.853, *p* = 0.014) dominated the diet, but their relative contribution declined markedly in the 20's. Notably, cannibalism by pikeperch was observed exclusively in the 20's (Figure 3).

**FIGURE 4:**
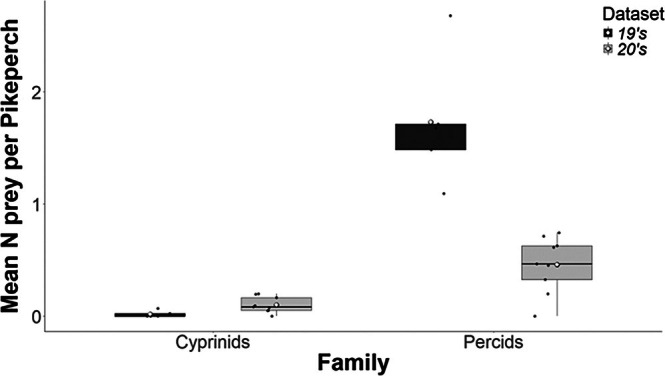
Mean prey per pikeperch, separated per family, between datasets. Median values (thick lines), upper and lower quartiles (boxes), maximum and minimum values (whiskers) and outliers (dots) are shown.

Dietary shifts were evident in the pikeperch prey composition at the family level. The proportion of cyprinids (bleak, bream, roach and silver bream) increased significantly in the 20's dataset (GLM quasi‐Poisson, df = 12, *R* ^2^ = 0.37, *t* value = 2.254, *p* = 0.044), whereas percids (perch, pikeperch and ruffe) decreased (GLM quasi‐Poisson, df = 12, *R* ^2^ = 0.70, *t* value = −5.612, *p* = 0.0001) (Figure 4).

**FIGURE 5:**
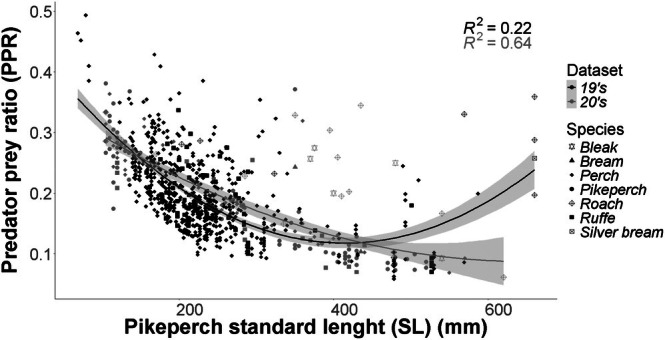
Predator–prey ratio (PPR) by standard length (SL) of pikeperch between the 19's and 20's datasets. The shapes represent the individual species data, the line represents the quadratic regression stratified on the dataset and the shaded area represents the respective confidence intervals of 95%.

The quadratic regression stratified on 19's/20's datasets revealed significant non‐linear relationships between predator SL and prey ratio (GLM: deviance explained = 94.3%, adjusted *R* ^2^ = 0.494). The 19's dataset exhibited a pronounced *U*‐shaped relationship with stronger curvature [quadratic coefficient = 1.9 × 10^−6^, 95% confidence interval (CI) (1.8 × 10^−6^, 2.2 × 10^−6^)] compared to the 20's [6.907 × 10^−7^, 95% CI (2.86 × 10^−7^, 1.1 × 10^−6^)). Size‐dependent patterns diverged markedly, with the 19's exhibiting steeper declines in prey ratio per mm SL [−0.00166, 95% CI (−0.001899, −0.001413)] vs. the 20's [−0.000896, 95% CI (−0.001082, −0.000709)] (Table 2). Pikeperch cannibalism was only recorded in the 20's dataset (Figure 5).

**TABLE 2:** Parameter estimates, standard errors (SE) and 95% confidence intervals (lower and upper limits) for the quadratic regression coefficients describing predator–prey size relationships separately in the 19's and 20's datasets.

		Estimate	SE	Lower 95% CI	Upper 95% CI
19's	A	0.4606000	0.0140700	0.4330228	0.4881772
	B	−0.0016560	0.0001240	−0.0018990	−0.0014130
	C	0.0000019	0.0000001	0.0000018	0.0000022
20's	A	0.3778300	0.0158200	0.3468228	0.4088372
	B	−0.0008958	0.0000950	−0.0010820	−0.0007096
	C	0.0000007	0.0000002	0.0000003	0.0000011

**FIGURE 6:**
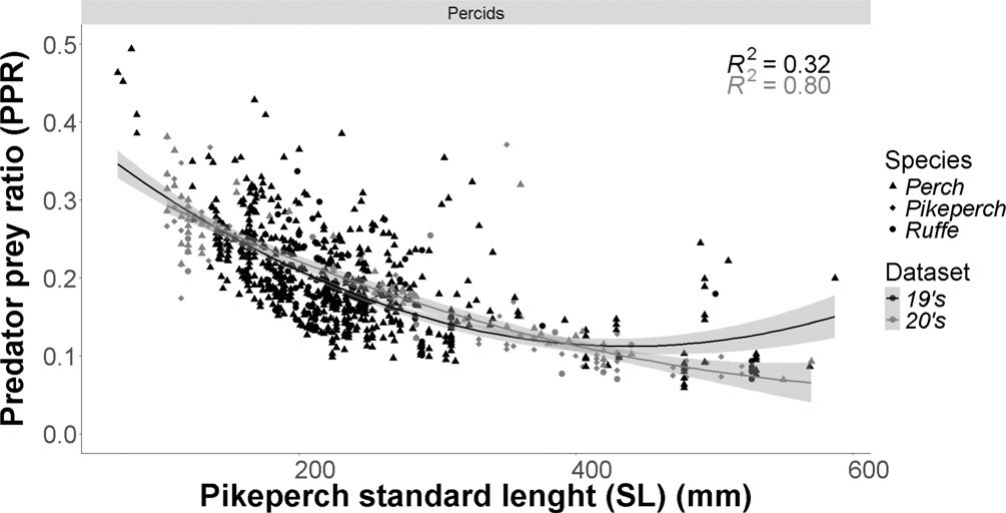
Predator–prey ratio (PPR) by standard length (SL) of pikeperch for the 19's and 20's datasets of the percid family. The shapes represent the individual species measures, the line represents the quadratic regression of the dataset and the shaded area represents the respective confidence intervals of 95%.

The analysis of percid prey revealed significant shifts in prey ratios across predator sizes (GLM: deviance explained = 95%, adjusted *R* ^2^ = 0.549). The 19's dataset showed stronger quadratic scaling [quadratic coefficient = 1.719 × 10^−6^, 95% CI (1.3 × 10^−6^, 2.1 × 10^−6^)] compared to the 20's [7.110 × 10^−7^, 95% CI (2.659 × 10^−7^, 1.1 × 10^−6^)]. Size‐dependent patterns diverged markedly, with the 19's exhibiting steeper declines in prey ratio per mm SL [−0.001506, 95% CI (−0.001709, −0.001303)] versus the 20's [−0.000969, 95% CI (−0.001231, −0.000707)] (Figure 6; Table 3).

**TABLE 3:** Parameter estimates, standard errors (SE) and 95% confidence intervals (lower and upper limits) for the quadratic regression coefficients describing percid prey size relationships separately in the 19's and 20's datasets.

		Estimate	SE	Lower 95% CI	Upper 95% CI
19's	a	0.4418710	0.0147300	0.4130002	0.4707418
	b	−0.0015060	0.0001037	−0.0017093	−0.0013027
	c	0.0000017	0.0000002	0.0000013	0.0000021
20's	a	0.3868590	0.0163500	0.3548130	0.4189050
	b	−0.0009692	0.0001335	−0.0012309	−0.0007075
	c	0.0000007	0.0000002	0.0000003	0.0000011

**FIGURE 7:**
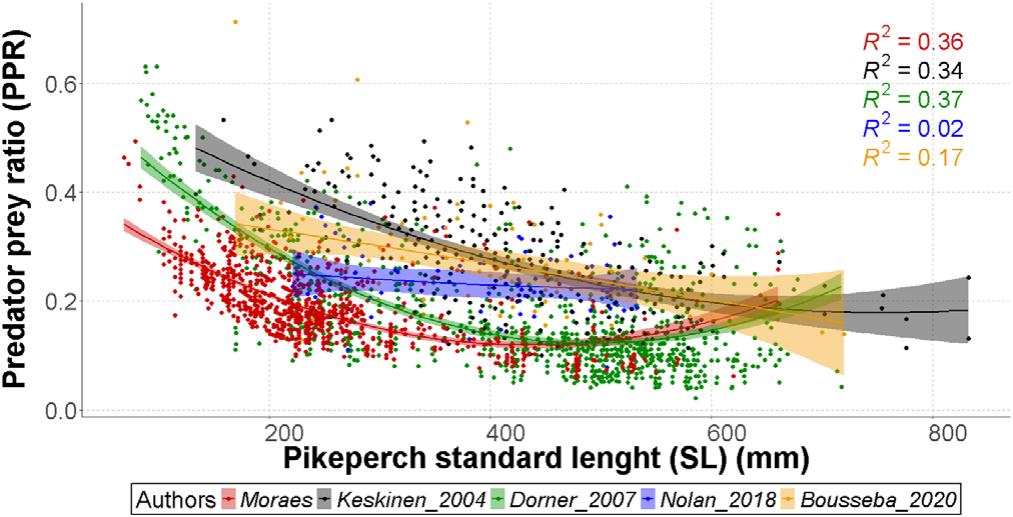
Predator–prey ratio (PPR) by standard length (SL) of pikeperch between our dataset and values extracted from published literature. The coloured dots represent individual measures, the line represents the quadratic regression of the data and the shaded area represents the 95% confidence interval.

The comparative analysis revealed divergent predator–prey size relationships across studies (GLM: deviance explained = 90.9%, adjusted *R* ^2^ = 52.6), with this study [quadratic coefficient = 1.643 × 10^−6^, 95% CI (1.345 × 10^−6^, 1.941 × 10^−6^)], Keskinen_2004 [7.854 × 10^−7^ (4.238 × 10^−7^, 1.147 × 10^−6^)] and Dorner_2007 [2.055 × 10^−6^ (1.840 × 10^−6^, 2.270 × 10^−6^)] exhibited significant *U*‐shaped patterns. In contrast, Nolan_2018 and Bousseba_2020 showed lower quadratic coefficient. Baseline prey ratios differed markedly among studies [Moraes: 0.432 (0.404, 0.459); Keskinen_2004: 0.626 (0.550, 0.701); Dorner_2007: 0.619 (0.590, 0.649); Nolan_2018: 0.292 (−0.005, 0.590); Bousseba_2020: 0.392, (0.266, 0.518)]. Bousseba_2020 [linear coefficient = −2.90 × 10^−4^ (−0.926 × 10^−3^, −0.346 × 10^−3^)] and Nolan_2018 [−2.30 × 10^−4^ (−1.864 × 10^−3^, −1.404 × 10^−3^)] showed shallowest size dependence between studies. Model diagnostics confirmed robustness (generalised cross‐validation = 0.00465) with the quadratic terms between studies (Figure 7; Table 4).

**TABLE 4:** Parameter estimates, standard errors (SE) and 95% confidence intervals (lower and upper limits) for the quadratic regression coefficients describing predator–prey size relationships obtained by different authors.

		Estimate	SE	Lower 95% CI	Upper 95% CI
Moraes	a	0.43166000	0.0141700	0.4038868	0.4594332
	b	−0.00143000	0.0000989	−0.0016238	−0.0012362
	c	0.00000164	0.00000015	0.0000013	0.0000019
Keskinen_2004	a	0.62552000	0.0385000	0.5500600	0.7009800
	b	−0.00119000	0.0001716	−0.0015263	−0.0008537
	c	0.00000079	0.00000018	0.0000004	0.0000011
Dorner_2007	a	0.61935000	0.0150500	0.5898520	0.6488480
	b	−0.00202000	0.0000852	−0.0021871	−0.0018529
	c	0.00000205	0.00000011	0.0000018	0.0000023
Nolan_2018	a	0.29244000	0.1516000	−0.0046960	0.5895760
	b	−0.00023000	0.0008341	−0.0018648	0.0014048
	c	0.00000018	0.00000109	−0.0000020	0.0000023
Bousseba_2020	a	0.39211000	0.0644800	0.2657292	0.5184908
	b	−0.00029000	0.0003247	−0.0009264	0.0003464
	c	−0.00000004	0.00000038	−0.0000008	0.0000007

**FIGURE 8:**
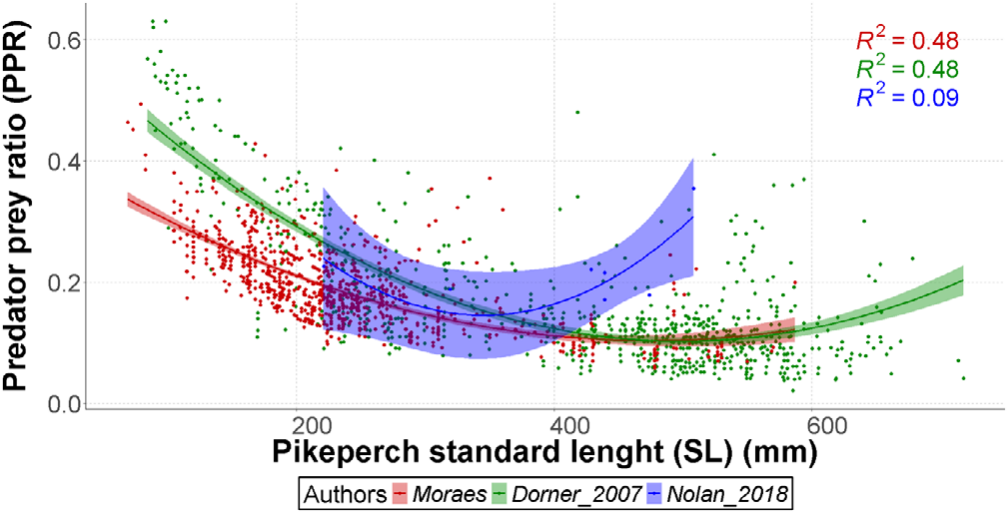
Predator–prey ratio (PPR) by standard length (SL) of pikeperch on our data and data extracted from other literature of the percid family. The different colour dots represent individual measures, the lines represent the quadratic regression of the data and the shaded area represents the 95% confidence interval.

The percid analysis between studies revealed significant change in prey ratios across all studies, with distinct quadratic relationships for this study [quadratic coefficient = 1.420 × 10^−6^, 95% CI (1.112, 1.728) × 10^−6^)], Dorner_2007 [2.113 × 10^−6^ (1.914, 2.312) × 10^−6^)] and Nolan_2018 [6.115 × 10^−6^ (0.788, 11.442) × 10^−6^)]. Model diagnostics indicated good fit (adjusted *R* ^2^ = 0.614), though Nolan_2018's wider CIs suggest greater uncertainty in its extreme estimates (Table 5; Figure 8).

**TABLE 5:** Parameter estimates, standard errors (SE) and 95% confidence intervals (lower and upper limits) for the quadratic regression coefficients describing predator – percid family prey size relationships between authors.

		Estimate	SE	Lower 95% CI	Upper 95% CI
Moraes	a	0.42286	0.0134400	0.3965176	0.4492024
	b	−0.00135	0.0000973	−0.0015407	−0.0011593
	c	0.00000142	0.00000016	0.0000011	0.0000017
Dorner_2007	a	0.62929	0.0134100	0.6030064	0.6555736
	b	−0.00211	0.0000776	−0.0022622	−0.0019578
	c	0.00000211	0.00000010	0.0000019	0.0000023
Nolan_2018	a	0.87342	0.3457000	0.1958480	1.5509920
	b	−0.00422	0.001982	−0.0081047	−0.0003353
	c	0.00000611	0.00000272	0.0000008	0.0000114

**FIGURE 9:**
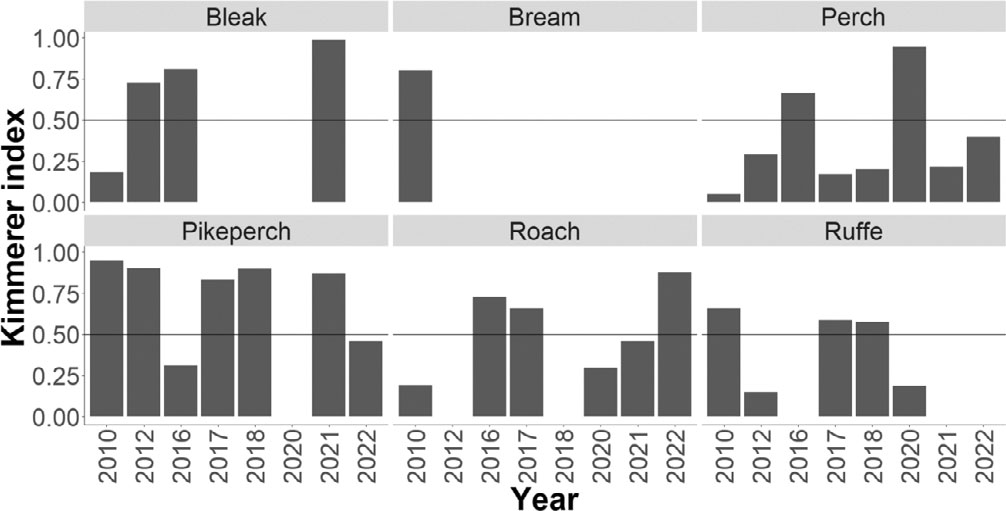
Bar plot of Kimmerer electivity by prey species from the 20's dataset, showing annual values. Gaps in the histogram indicate years when the species was not detected in pikeperch stomach samples. Values >0.5 indicate positive selection, whereas values <0.5 indicate negative selection.

The electivity index analysis revealed strong positive selection for cannibalism by pikeperch (Figure 9). Although perch represented the most frequent prey item in our study, this likely reflected its high abundance in the reservoir rather than active selection, as indicated by low electivity values. Comparative analysis suggested lower preference for perch relative to both pikeperch and bleak. Among cyprinids, bleak was the only species showing consistently positive electivity, though its occurrence in pikeperch diets demonstrated notable interannual variation.

## 4 DISCUSSION

Our long‐term study confirmed the hypothesis of significant temporal transformations in the trophic ecology of pikeperch in Lipno Reservoir, reflecting broader limnological changes characteristic of ageing impoundments. The data demonstrate three fundamental shifts: first, a substantial 30% reduction in mean prey consumption per predator between the 19's and 20's; second, increased diet diversity, which may indicate the need to consume less‐preferred prey; and third, the emergence of significant cannibalism, which occurred in 39.78% of total predation during the 20's compared to 0% in the 19's samples.

As an opportunistic predator, pikeperch preferentially targets the most abundant prey species within its habitat (Kangur et al., 2007; Ribeiro et al., 2021). During the 19's, optimal conditions supported high pikeperch densities (Jůza et al., 2023; Vostradovska & Vostradovsky, 1986). However, prey composition varies seasonally in response to fluctuations in prey availability in the waterbody (Bousseba et al., 2020; Kangur et al., 2007; Kangur & Kangur, 1998). In the 20's, pikeperch in Lipno primarily consumed YOY fish, likely due to two factors: the relatively large size of YOY prey (40–70 mm SL) during late‐summer sampling, and the scarcity of older, larger prey fish due to limited survival from the previous year. This heavy reliance on YOY prey mirrors patterns observed in Lake Balaton, Hungary (Specziár, 2011). Strong preference to YOY, especially at the 20's dataset, weakened a relationship between predator size and prey size in pikeperch. Consequently, Lipno pikeperch exhibited significantly lower PPR than other studied populations. This trend aligns with optimal foraging theory (Bolnick et al., 2002; Stephens & Krebs, 1986), as smaller prey offer lower handling costs and favourable energy returns relative to capture effort of bigger preys. However, the best fit of SL/PPR relationships was achieved using *U*‐shaped models that suggest that the largest pikeperch consumed increased fish prey. Also, pikeperch in other systems may feed on larger prey because of greater availability and more calories per prey item.

Pikeperch in Lipno Reservoir had a relatively low proportion of stomachs without fish compared to findings reported by other authors (this study: 31.54%, Keskinen_2004: 42%, Specziár _2011: 68.7%, Nolan_2018: 54.4%, Bousseba_2020: 39%). A larger proportion of empty stomachs in other localities could be consistent with a higher PPR (Figure 7), as pikeperch foraging on larger prey may exhibit longer periods with empty stomachs. Empty stomachs can create some bias within the results, as this can be influenced by capturing the fish outside its feeding time or prey regurgitation due to stress induced by fishing gear (Vignon & Dierking, 2011; Zhao et al., 2020). However, at the moment of this publication, correction calculations for this specific problem have not been found and Lipno data seem to be less biased than others in this respect.

As cannibalism starts showing in the 20's dataset quite strongly, it also indicates that the prey abundance in the reservoir may be lower than in the 19's. Cannibalism is a prevalent phenomenon in pikeperch populations, characterized by larger, early‐hatched YOY (0+) fish preying on the smaller, late‐hatched fish (Tesfaye et al., 2025). This is normally triggered by the low amount of other prey in the waterbody due to competition for food or predatory pressure from larger predators (Mehner et al., 1996). In pikeperch, cannibalism is further intensified by strong differences in year classes, where elevated fry densities facilitate size‐dependent intraspecific predation, driven by ontogenetic growth variation within same cohorts (Frankiewicz et al., 1999; Lappalainen et al., 2006). Jůza et al. (2023) observed a pattern where lower pikeperch fry densities in the reservoir were associated with greater SLs. The time frame of this study aligns with our 20's dataset, when cannibalism occurred and can be considered confirmation for the presence of both intracohort and intercohort cannibalism in this population. High level of cannibalism was also observed in Hungary (Specziár, 2011). During this study, pikeperch showed quite a high index value for cannibalism for both pikeperch and Volga pikeperch (*Sander volgensis*). In localities with larger prey (high PPR), no cannibalism was reported (Keskinen & Marjomäki, 2004; Nolan & Britton, 2018).

According to our data, perch was an important prey for pikeperch in both time periods and in all ranges of pikeperch size. Perch predation was dominant in the 19's, even considering large proportion of cyprinid YOY in these years (Vostradovska & Vostradovsky, 1986; Vostradovsky, 1965). This pattern suggests active prey selection despite alternative prey availability, potentially driven by higher catchability of perch in littoral habitats or reduced predation risk when targeting solitary perch versus schooling cyprinids (Turesson & Brönmark, 2007). The data collected in the 20's showed a shift in pikeperch prey to more diverse prey. In both periods, the PPR exhibited a clear declining slope; however, the 19's data showed an increase in PPR at larger pikeperch sizes. This was not observed in 20's data, suggesting that larger prey sizes were scarce in the reservoir. The scarcity of larger prey fish, or the low overall abundance of suitably sized fish in the reservoir, is likely limiting the pikeperch's foraging options. Especially in the early 2000s, pikeperch seemed to be food‐limited, exhibiting slower growth (Tesfaye et al., 2023), with only partial recovery by 2015 compared to historical levels (Vostradovska & Vostradovsky, 1964, 1986).

The apparent dominance of YOY fish in pikeperch diets during the 20's likely reflects a combination of suboptimal prey densities and opportunistic foraging behaviour. The results of prey electivity revealed interesting patterns. Although YOY prey (particularly perch) constituted the majority of stomach contents, electivity analysis revealed no strong active selection for these prey items. This suggests that their dietary prevalence was driven primarily by high relative abundance rather than preferential predation – a pattern contrasting with the 19's data (though direct electivity comparisons are not possible by data insufficiency – absence of information on juvenile fish community). This effect, wherein a primary prey resource is large consumed in numbers, but not actively selected by the predator, can be explained by two factors: (i) prey size suitability, YOY fish (40–70 mm SL) represented the optimal prey size class for most pikeperch in the reservoir, balancing energy content and capture efficiency (Bolnick et al., 2002); (ii) Passive versus active selection, pikeperch may exploit locally abundant YOY prey without exhibiting classic electivity when overall prey diversity is low (Specziár, 2011). The species' renowned dietary flexibility allows this adaptive response to changing prey communities, though the nutritional trade‐offs of sustained YOY predation may explain the observed growth reductions (Tesfaye et al., 2023).

## 5 CONCLUSIONS

The changes in Lipno Reservoir community were reflected in pikeperch stomach content. The average prey count per stomach had a loss of 30.12% from 1966–1970 (1.66 fish) to 2010–2022 (1.16 fish). The diet was heavily dominated by the common perch (86.93% in the 19's total prey, 41.24% in the 20's total prey). Although percids remain the dominant prey, elevated cannibalism was recorded with our data (0% in the 19's total prey, 39.78% in the 20's total prey). Also, the proportion of cyprinid fish increased significantly. Especially in the 20's, larger pikeperch had a lower PPR, meaning they consumed proportionally smaller prey as they grew. Our results showed the shallowest predator–prey ratio among other studies, suggesting that YOY fish as the staple prey for all pikeperch cohorts in Lipno Reservoir, the PPR range in Lipno still overlapped with that of larger individuals reported by other authors. This study is important for explaining the changes in pikeperch abundance with respect to resources availability, cannibalism and potential competition with other predators. It also underlines the need for sound information on real composition of forage fish base to predict predator behaviour and prosperity. Another very important point is the further refinement of stomach content methods: generally active immediate samples should be preferred to passive gear samples like gillnets. Immediate samples would be much more valuable for deeper insight of feeding bioenergetics. However, the utilization of gillnet samples for orientation feeding analysis represents a good use of the fish killed anyway by destructive approach. Also, applying this analysis to other waterbodies of different ages and productivities level would test the generality of these predation patterns and help build a model of pikeperch trophic ecology across environmental gradients. In summary, this study reveals a pikeperch population adapting to significant environmental change through dramatic dietary shifts.

## AUTHOR CONTRIBUTIONS

Karlos Ribeiro de Moraes, Million Tesfaye: conceptualization, investigation, data curation, formal analysis, methodology, software, validation, visualization, writing – original draft, writing – review and editing. Petr Blabolil, Tomáš Jůza, Allan T. Souza, Marek Šmejkal, Marie Prchalová, Mojmír Vašek: investigation, writing – review and editing. Luboš Kočvara, Milan Muska, Radka Symonová and Milan Říha: writing – review and editing and investigation. Katerina Soukalová, D. Bartoň, Michal Tuser, Zuzana Sajdlová, Vladislav Draštík and Jakub Brabec: investigation. Marek Brabec: formal analysis, methodology, writing – review and editing. Jan Kubečka: conceptualization, funding acquisition, investigation, methodology, project administration, resources, supervision, writing – review and editing.
